# Prognostic value of blood glucose trajectories in critically ill patients with intracerebral hemorrhage: A retrospective cohort study

**DOI:** 10.1371/journal.pone.0342745

**Published:** 2026-02-24

**Authors:** Huan Zuo, Xin Zuo, Yaxin Zhang, Weihong Zheng

**Affiliations:** 1 Department of Acupuncture, Heilongjiang University of Chinese Medicine, Harbin, China; 2 Department of Neurology, Xiamen Humanity Hospital, Fujian Medical University, Xiamen, Fujian, China; Fukuoka University, JAPAN

## Abstract

**Background:**

Abnormal glucose regulation is common in critically ill patients with intracerebral hemorrhage (ICH), but the prognostic relevance of early glucose patterns remains unclear. We aimed to identify early blood glucose trajectories in ICU patients with ICH and evaluate their association with short-term mortality.

**Methods:**

Adult patients with ICH were identified from the MIMIC-IV database. Latent class analysis was applied to blood glucose measurements obtained within the first 36 hours after ICU admission to identify distinct glucose trajectory classes. Cox proportional hazards models were used to assess the association between glucose trajectories and 28-day in-hospital mortality with multivariable adjustment. A 36-hour landmark analysis and sensitivity analyses including patients with ICU length of stay <36 hours were conducted to evaluate robustness.

**Results:**

A total of 1,978 patients were classified into three glucose trajectory classes: stable normoglycemia, a U-shaped pattern with initial severe hyperglycemia, and an inverted U-shaped pattern with marked glycemic fluctuation. Compared with the stable group, both unfavorable trajectory classes were associated with significantly higher 28-day in-hospital mortality after full adjustment (*P* <  0.05). These associations were consistent across landmark and sensitivity analyses. Kaplan–Meier analyses demonstrated significant survival differences among trajectory classes, and subgroup analyses suggested effect modification by age and diabetes status.

**Conclusions:**

Early blood glucose trajectories within the first 36 hours after ICU admission are independently associated with 28-day in-hospital mortality in patients with ICH. Dynamic glucose patterns may provide additional prognostic value for early risk stratification.

## 1. Introduction

Intracerebral hemorrhage (ICH) is an acute hemorrhagic stroke caused by the rupture of blood vessels within the brain parenchyma, mainly due to hypertensive small vessel disease and cerebral amyloid angiopathy. Its incidence is about 24.6 per 100,000 people, with higher rates in men, the elderly, and especially in Asian populations. ICH has a high mortality rate—approximately 40% at 30 days and 50% at one year—with only about 20% of patients regaining independence within six months. Hypertension is the primary risk factor, and elevated blood pressure and heart rate on admission are associated with more severe illness and poorer prognosis. ICH is a serious condition requiring early intervention [1–3].

Blood glucose trajectory, representing the dynamic fluctuations in blood glucose over time, has become an important biomarker in critical care. It provides a comprehensive reflection of metabolic status by capturing glycemic trends. This trajectory reflects the interaction between stress responses, insulin regulation, and inflammation, which are crucial in acute brain injury. Studies show that blood glucose variability is closely linked to disease trajectories in traumatic brain injury patients, suggesting its potential in prognosis assessment and therapeutic monitoring [4,5].

Beyond ICH, blood glucose trajectory have been extensively studied in various critical illnesses, such as sepsis, myocardial infarction, and ischemic stroke, where abnormal glycemic patterns have been linked to worse outcomes [6–8]. These findings suggest that glucose variability may contribute to secondary injury cascades, including oxidative stress and neuroinflammation [9–11]. However, in patients with ICH, the prognostic significance of blood glucose trajectory remains unclear, with limited and inconsistent data available.

We utilized the Medical Information Mart for Intensive Care IV (MIMIC-IV) database to examine the association between blood glucose trajectory and clinical outcomes in patients with ICH. Our study aims to elucidate the prognostic value of dynamic glycemic patterns in this population, which may inform more personalized glucose management and improve clinical decision-making.

## 2. Materials and methods

### 2.1. Data source

This retrospective cohort study utilized data from the Medical Information Mart for Intensive Care IV (MIMIC-IV, version 3.1) database, a large-scale, publicly accessible, de-identified critical care dataset [12]. MIMIC-IV contains comprehensive clinical information on patients admitted to intensive care units at Beth Israel Deaconess Medical Center from 2008 to 2022, including demographics, vital signs, laboratory tests, medication records, and clinical outcomes. The creation of the MIMIC database was approved by the Institutional Review Boards (IRB) of Beth Israel Deaconess Medical Center (Boston, MA, USA) and Massachusetts Institute of Technology (Cambridge, MA, USA), with a waiver of informed consent granted due to the retrospective nature of data collection and complete anonymization of all medical records. The current data analysis was approved by the Beth Israel Deaconess Medical Center IRB (Approval number：2001P-001699/14). All research methods were performed in accordance with relevant guidelines and regulations, including the Declaration of Helsinki and MIMIC database usage agreements.The database serves as a robust platform for observational studies aimed at improving critical care practices. One of the authors, Huan Zuo, fulfilled all necessary training and credentialing requirements for accessing the MIMIC-IV database and was responsible for data extraction and management for the present study (Record ID: 65378168 (for HZ)).

### 2.2. Inclusion and exclusion criteria

**Fig 1:**
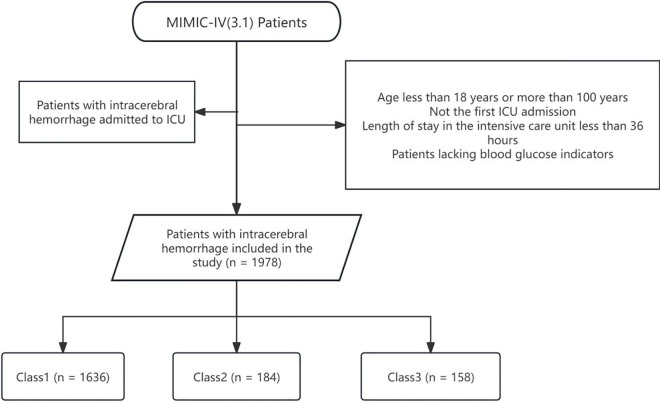
Flowchart of patient selection and classification based on blood glucose trajectories among patients with intracerebral hemorrhage.

Patients with a diagnosis of ICH were identified from the MIMIC-IV version 3.1 database using standardized codes from the International Classification of Diseases, 9th and 10th Revisions (ICD-9 and ICD-10). To assess the prognostic implications of glucose trajectory patterns, several exclusion criteria were implemented to ensure both the integrity of the data and clinical relevance: (a) age < 18 years or >100 years; (b) ICU length of stay <36 hours; (c) not the first ICU admission; (d) absence of blood glucose measurements recorded at least every 12 hours during the first 36 hours following ICU admission (Fig 1).

### 2.3. Data collection

We extracted comprehensive clinical data from the MIMIC-IV database for all eligible patients. The collected variables included demographic characteristics [age, gender, race], vital signs [heart rate, systolic blood pressure (SBP), diastolic blood pressure (DBP), mean blood pressure (MBP), respiratory rate, temperature, oxygen saturation (SpO₂)], and laboratory indices. Laboratory data comprised coagulation parameters [international normalized ratio (INR), prothrombin time (PT), partial thromboplastin time (PTT)], electrolytes [sodium, potassium, chloride, bicarbonate, calcium, anion gap], renal function markers [creatinine, blood urea nitrogen (BUN)], and hematological parameters [white blood cell count (WBC), red blood cell count (RBC), hemoglobin, hematocrit, mean corpuscular volume (MCV), mean corpuscular hemoglobin (MCH), red cell distribution width (RDW), platelet count].In addition, clinical severity and prognostic scores [Sequential Organ Failure Assessment (SOFA), Simplified Acute Physiology Score II (SAPS II), Glasgow Coma Scale (GCS), Charlson Comorbidity Index] were recorded. Comorbidities and complications were documented as dichotomous variables acute kidney injury (AKI), intraventricular hemorrhage, myocardial infarction, renal disease, liver disease, sepsis, respiratory failure, congestive heart failure, peripheral vascular disease, dementia, chronic pulmonary disease, rheumatic disease, peptic ulcer disease, diabetes, hypertension.

Treatment-related variables included [mechanical ventilation, continuous renal replacement therapy (CRRT), corticosteroids, insulin, dextrose infusion]. Outcome measures comprised [length of hospital stay within 28 days, hospital survival, 28-day mortality].

Data were extracted using PostgreSQL software. Baseline laboratory and physiological variables used as covariates were obtained from measurements recorded during the first 24 hours after ICU admission. In contrast, blood glucose measurements used for glycemic trajectory modeling were extracted from the first 36 hours after ICU admission, as described below.

### 2.4. Outcome

The primary outcome of this study was all-cause in-hospital mortality within 28 days in patients with intracerebral hemorrhage. Follow-up began at ICU admission and continued until in-hospital death or discharge, whichever occurred first.

### 2.5. Latent Growth Mixture Modeling (LGMM)

Blood glucose values were recorded at 12, 24, and 36 hours following ICU admission. A Latent Growth Mixture Model (LGMM) was applied to identify distinct blood glucose trajectory patterns within the first 36 hours of ICU stay. The LGMM was implemented using the “lcmm” package in R to identify trajectory classes of blood glucose levels. LGMM assumes that the study population comprises several unobserved (latent) subgroups, each following a distinct class-specific trajectory characterized by unique average trends over time.

Determining the optimal number of trajectory classes is a key step in LGMM analysis. Quadratic growth models with one to seven trajectory classes were constructed [13]. Model selection was guided by a combination of statistical and practical criteria: (1) lower values of Akaike Information Criterion (AIC), Bayesian Information Criterion (BIC), and sample-size adjusted BIC indicate better model fit; (2) higher log-likelihood and entropy values were preferred, with entropy ≥0.7 considered acceptable; (3) each trajectory class was required to comprise at least 1% of the total sample; (4) the average posterior probability for each class was required to be ≥ 70% to ensure classification accuracy; and (5) model parsimony and clinical interpretability were considered in the final model selection.

### 2.6. Statistical analysis

Statistical analyses were performed using R software. Continuous variables were expressed as mean ± standard deviation or median (interquartile range), and categorical variables as counts and percentages. Group differences were tested with one-way ANOVA or Kruskal–Wallis test for continuous data, and Chi-square or Fisher’s exact test for categorical data.

LGMM identified blood glucose trajectory classes within the first 36 hours of ICU admission. The best model was chosen based on AIC, BIC, entropy, and class size. Variables with less than 15% missing data were imputed using random forest under the missing-at-random assumption; variables with more missing data were excluded from multivariate analysis. Cox regression was used to analyze the association between blood glucose trajectories and in-hospital mortality. Three models were constructed: unadjusted, adjusted for age, sex, and race, and further adjusted for variables with *P* < 0.05 in univariate analysis. Each trajectory class was used as the reference for pairwise comparisons.

To address potential survivor bias and immortal time bias related to the exclusion of patients with short ICU stays, we performed a 36-hour landmark analysis and a sensitivity analysis. For the landmark analysis, patients with sufficient glucose measurements within the first 36 hours after ICU admission to allow assignment of glucose trajectory groups were identified, and only those who were alive and still hospitalized at 36 hours after ICU admission were included. Early glucose trajectory groups were defined using glucose measurements obtained at approximately 12, 24, and 36 hours after ICU admission and were treated as fixed exposures determined before the landmark time. Follow-up started at 36 hours after ICU admission and continued until in-hospital death, discharge, or day 28 after ICU admission, whichever occurred first, using Cox proportional hazards models with the same covariate adjustment as in the primary analysis. In addition, a sensitivity analysis was conducted by including all eligible patients with sufficient glucose measurements at 12, 24, and 36 hours, regardless of ICU length of stay. In this analysis, follow-up began at ICU admission and extended to in-hospital death, discharge, or day 28, and multivariable Cox models identical to those in the primary analysis were applied to assess the robustness of the findings.

Kaplan–Meier curves and log-rank tests were used to compare 28-day survival. Subgroup analyses tested associations across age, sex, and major comorbidities. A p-value < 0.05 was considered statistically significant. Hazard ratios (HR) and 95% confidence intervals (CI) were reported.

## 3. Result

### 3.1. Identification and characterization of blood glucose trajectory classes

Latent class models with one to seven classes were evaluated to identify blood glucose trajectory patterns in ICU patients with intracerebral hemorrhage. Although model fit improved with more classes, as indicated by decreasing AIC and BIC values, the three-class model was selected as optimal due to its balance of fit and simplicity. This model had relatively low AIC and BIC, the highest relative entropy (0.895), indicating clear and stable classification, and a reasonable class distribution with 82.7% in the largest class and 17.3% in the smaller classes. The three-class model demonstrated high classification accuracy, significant trajectory distinctions, and minimal fitting error, making it suitable for further analysis and clinical application (Table 1).

**Table 1:** Fit indices of latent class models and class distributions of blood glucose trajectories in relation to 28-day prognosis of ICU patients with intracerebral hemorrhage.

G	loglik	AIC	BIC	Relative entropy	%class 1	%class 2	%class 3	%class 4	%class 5	%class 6	%class 7
1	−31100.07	62214.14	62253.26	1	100						
2	−30493.4	61010.79	61077.87	0.763	76.79	23.21					
3	−30168.62	60371.24	60466.27	0.895	82.71	9.3	7.99				
4	−29950.88	59945.77	60068.74	0.817	75.03	3.24	5.16	16.58			
5	−29837.88	59729.76	59880.69	0.749	39.69	12.89	39.43	4.95	3.03		
6	−29840.24	59744.48	59923.36	0.753	31.6	12.54	43.63	2.17	3.34	6.72	
7	−29725.81	59525.62	59732.44	0.704	23.46	34.68	20.73	9.2	2.17	3.74	6.02

G: The number of classes in the model.

loglik: The log-likelihood value.

AIC: Akaike Information Criterion.

BIC: Bayesian Information Criterion.

Relative entropy: A measure of the classification effectiveness of the model.

**Fig 2:**
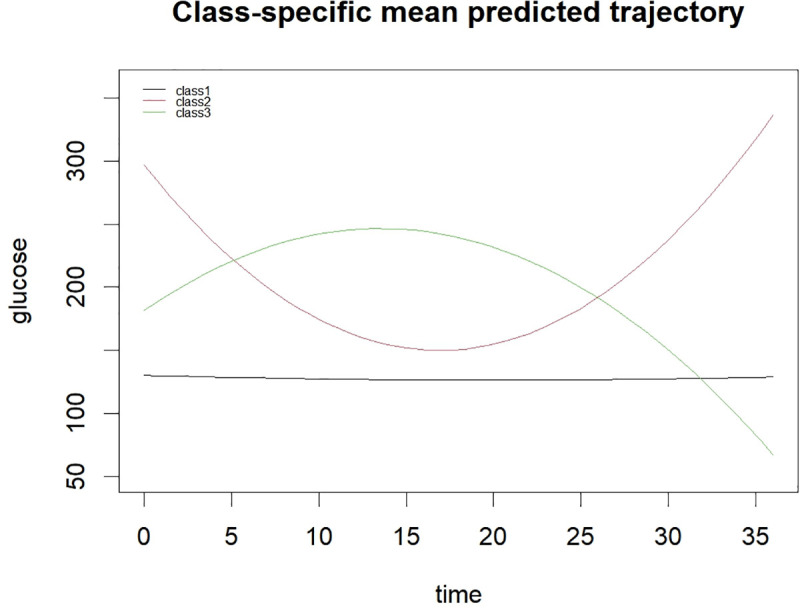
Mean predicted blood glucose trajectories for three latent classes. This figure shows the time-varying blood glucose levels in ICU patients with intracerebral hemorrhage for three latent classes. Class 1 exhibits relatively stable and low glucose levels, Class 2 shows a U-shaped trajectory with an initial decrease followed by an increase, and Class 3 presents an inverted U-shaped trajectory with an initial increase followed by a decrease.

Fig 2 shows the mean predicted blood glucose trajectories for the three latent classes. Class 1 maintains a stable glucose level around 100–120 mg/dL over 36 hours. Class 2 follows a U-shaped pattern, starting high (~300 mg/dL), dropping to about 150 mg/dL, then rising again. Class 3 displays an inverted U-shape, rising from ~180 mg/dL to a peak near 270 mg/dL before declining to around 50 mg/dL. These distinct patterns highlight the heterogeneity of glycemic responses in patients.

### 3.2. Clinical characteristics and prognostic impact of blood glucose trajectories

A total of 1,978 patients with intracerebral hemorrhage were classified into three blood glucose trajectory groups. Race distribution differed significantly among groups (*P* = 0.004), whereas age and sex were comparable. Patients in Class 2 and Class 3 showed higher heart rate and respiratory rate (*P* < 0.05) and had significantly worse laboratory profiles, including higher creatinine, blood urea nitrogen, white blood cell counts, and anion gap with lower bicarbonate levels (all *P* < 0.001). Disease severity scores were significantly higher in Class 2 and Class 3 (*P* < 0.05). Several comorbidities, particularly renal disease, sepsis, and diabetes, were more prevalent in these groups, with diabetes markedly more common (*P* < 0.001). Insulin and dextrose infusion were used more frequently in Class 2 and Class 3 (*P* < 0.001) (Table 2).

**Table 2:** Clinical characteristics and prognosis of blood glucose trajectories in patients with Intracerebral hemorrhage.

Variables	Total (n = 1978)	Class 1 (n = 1636)	Class 2 (n = 184)	Class 3 (n = 158)	*P*
Age	69.45 (57.95, 79.95)	69.66 (57.88,80.24)	67.22 (58.14,77.56)	68.62 (59.37,78.46)	0.312
Gender, n (%)					0.169
Female	904 (45.70)	763 (46.64)	78 (42.39)	63 (39.87)	
Man	1074 (54.30)	873 (53.36)	106 (57.61)	95 (60.13)	
Race, n (%)					0.004
White	1146 (57.94)	963 (58.86)	91 (49.46)	92 (58.23)	
Black	214 (10.82)	164 (10.02)	35 (19.02)	15 (9.49)	
Others	618 (31.24)	509 (31.11)	58 (31.52)	51 (32.28)	
**Vital signs**					
Heart Rate, bpm	81.50 (71.00, 94.00)	81.00 (70.00,93.00)	84.00 (72.00,96.00)	84.50 (76.00,96.00)	0.006
SBP, mmHg	137.00 (122.00, 151.00)	137.00 (122.00,150.00)	136.50 (121.75,155.00)	137.00 (119.25,154.75)	0.798
DBP, mmHg	74.00 (64.00, 86.00)	74.00 (64.00,86.00)	70.00 (60.00,86.25)	71.50 (62.00,84.00)	0.061
MBP, mmHg	91.00 (81.00, 102.75)	92.00 (81.00,102.25)	88.00 (77.75,103.25)	90.00 (78.00,102.00)	0.246
Resp Rate, bpm	18.00 (15.50, 22.00)	18.00 (15.00,22.00)	19.00 (16.00,22.00)	19.00 (16.00,21.00)	0.031
Temperature, °C	36.83 (36.52, 37.11)	36.83 (36.56,37.11)	36.70 (36.44,37.00)	36.83 (36.52,37.11)	0.008
SpO_2_, %	98.00 (96.00, 100.00)	98.00 (96.00,100.00)	98.50 (97.00,100.00)	98.00 (96.00,100.00)	0.186
**Laboratory index**					
Sodium, mEq/L	139.00 (137.00, 142.00)	139.00 (137.00,142.00)	139.00 (137.00,142.25)	138.50 (136.00,142.00)	0.361
Potassium, mEq/L	3.90 (3.60, 4.30)	3.90 (3.60,4.30)	4.00 (3.60,4.40)	4.00 (3.70,4.40)	0.068
Creatinine, mEq/L	0.90 (0.70, 1.10)	0.90 (0.70,1.10)	1.10 (0.80,1.50)	1.00 (0.80,1.30)	<0.001
WBC, K/uL	10.50 (8.10, 13.50)	10.30 (8.00,13.20)	11.75 (9.00,15.30)	11.65 (8.75,14.30)	<0.001
RDW, %	13.90 (13.10, 14.90)	13.80 (13.10,14.80)	13.90 (13.10,15.00)	14.00 (13.20,15.40)	0.324
RBC, M/uL	4.07 (3.60, 4.52)	4.08 (3.63,4.52)	4.11 (3.55,4.55)	4.02 (3.48,4.53)	0.537
Platelet, K/uL	208.00 (160.00, 259.00)	206.50 (161.00,257.00)	210.50 (150.00,262.25)	215.50 (167.25,293.75)	0.201
Hemoglobin, g/dL	12.20 (10.80, 13.50)	12.20 (10.90,13.60)	11.90 (10.50,13.50)	11.85 (10.20,13.40)	0.067
Hematocrit, %	36.75 (32.82, 40.40)	36.90 (33.10,40.50)	36.15 (32.08,40.62)	35.45 (31.33,39.90)	0.024
MCV, fL	30.20 (28.70, 31.60)	30.25 (28.80,31.70)	29.80 (28.40,30.90)	29.70 (28.30,31.30)	<0.001
MCH, pg	91.00 (87.00, 95.00)	91.00 (87.00,95.00)	90.00 (86.00,94.00)	90.00 (85.00,94.00)	0.002
INR	1.10 (1.10, 1.30)	1.10 (1.10,1.30)	1.10 (1.08,1.30)	1.20 (1.10,1.30)	0.186
PT	12.60 (11.70, 14.10)	12.60 (11.70,14.10)	12.45 (11.50,13.90)	13.00 (11.90,14.07)	0.304
PTT	28.30 (25.80, 31.40)	28.60 (25.90,31.50)	27.90 (24.50,31.33)	27.30 (25.22,30.73)	0.009
Chloride, mEq/L	104.00 (101.00, 107.00)	104.00 (101.00,106.00)	103.00 (100.00,107.00)	103.00 (100.00,107.00)	0.807
Aniongap, mEq/L	14.00 (12.00, 16.00)	14.00 (12.00,16.00)	15.00 (13.00,18.00)	15.00 (13.00,18.00)	<0.001
Bicarbonate, mEq/L	23.00 (21.00, 25.00)	23.00 (21.00,25.00)	23.00 (20.00,25.00)	22.00 (20.00,24.00)	<0.001
Calcium, mEq/L	8.80 (8.30, 9.20)	8.80 (8.30,9.20)	8.80 (8.30,9.20)	8.80 (8.30,9.17)	0.599
BUN, mg/dL	16.00 (12.00, 23.00)	16.00 (12.00,22.00)	20.00 (14.00,31.25)	18.00 (13.00,28.75)	<0.001
**Score**					
SOFA	1.00 (0.00, 2.00)	1.00 (0.00,2.00)	1.00 (0.00,2.00)	0.00 (0.00,2.00)	0.015
SAPSII	33.00 (26.00, 41.00)	33.00 (26.00,41.00)	35.00 (30.00,43.00)	36.00 (28.00,44.75)	<0.001
GCS	14.00 (11.00, 15.00)	14.00 (11.00,15.00)	14.00 (10.00,15.00)	14.00 (10.00,15.00)	0.635
Charlson Comorbidity Index	6.00 (4.00, 8.00)	6.00 (4.00,8.00)	7.00 (4.00,8.00)	6.00 (5.00,8.00)	<0.001
**Complication**					
AKI, n (%)					0.354
No	348 (17.59)	297 (18.15)	28 (15.22)	23 (14.56)	
Yes	1630 (82.41)	1339 (81.85)	156 (84.78)	135 (85.44)	
Intraventricular Hemorrhage, n (%)					0.985
No	1620 (81.90)	1341 (81.97)	150 (81.52)	129 (81.65)	
Yes	358 (18.10)	295 (18.03)	34 (18.48)	29 (18.35)	
Myocardial Infarct, n (%)					0.021
No	1790 (90.50)	1494 (91.32)	158 (85.87)	138 (87.34)	
Yes	188 (9.50)	142 (8.68)	26 (14.13)	20 (12.66)	
Renal Disease, n (%)					<0.001
No	1697 (85.79)	1432 (87.53)	138 (75.00)	127 (80.38)	
Yes	281 (14.21)	204 (12.47)	46 (25.00)	31 (19.62)	
Liver Disease, n (%)					0.708
No	1845 (93.28)	1529 (93.46)	171 (92.93)	145 (91.77)	
Yes	133 (6.72)	107 (6.54)	13 (7.07)	13 (8.23)	
Sepsis, n (%)					0.002
No	1020 (51.57)	873 (53.36)	78 (42.39)	69 (43.67)	
Yes	958 (48.43)	763 (46.64)	106 (57.61)	89 (56.33)	
Respiratory Failure, n (%)					<0.001
No	1306 (66.03)	1111 (67.91)	107 (58.15)	88 (55.70)	
Yes	672 (33.97)	525 (32.09)	77 (41.85)	70 (44.30)	
Congestive Heart Failure, n (%)					0.002
No	1660 (83.92)	1390 (84.96)	138 (75.00)	132 (83.54)	
Yes	318 (16.08)	246 (15.04)	46 (25.00)	26 (16.46)	
Peripheral Vascular Disease, n (%)					0.886
No	1833 (92.67)	1518 (92.79)	170 (92.39)	145 (91.77)	
Yes	145 (7.33)	118 (7.21)	14 (7.61)	13 (8.23)	
Dementia, n (%)					0.708
No	1850 (93.53)	1527 (93.34)	173 (94.02)	150 (94.94)	
Yes	128 (6.47)	109 (6.66)	11 (5.98)	8 (5.06)	
Chronic Pulmonary Disease, n (%)					0.741
No	1705 (86.20)	1406 (85.94)	160 (86.96)	139 (87.97)	
Yes	273 (13.80)	230 (14.06)	24 (13.04)	19 (12.03)	
Rheumatic Disease, n (%)					0.519
No	1927 (97.42)	1596 (97.56)	179 (97.28)	152 (96.20)	
Yes	51 (2.58)	40 (2.44)	5 (2.72)	6 (3.80)	
Peptic Ulcer Disease, n (%)					0.038
No	1962 (99.19)	1624 (99.27)	184 (100.00)	154 (97.47)	
Yes	16 (0.81)	12 (0.73)	0 (0.00)	4 (2.53)	
Diabetes, n (%)					<0.001
No	1397 (70.63)	1298 (79.34)	54 (29.35)	45 (28.48)	
Yes	581 (29.37)	338 (20.66)	130 (70.65)	113 (71.52)	
Hypertension, n (%)					0.144
No	726 (36.70)	585 (35.76)	78 (42.39)	63 (39.87)	
Yes	1252 (63.30)	1051 (64.24)	106 (57.61)	95 (60.13)	
**Treatment**					
Ventilator, n (%)					0.078
No	508 (25.68)	436 (26.65)	36 (19.57)	36 (22.78)	
Yes	1470 (74.32)	1200 (73.35)	148 (80.43)	122 (77.22)	
CRRT, n (%)					0.122
No	1928 (97.47)	1598 (97.68)	175 (95.11)	155 (98.10)	
Yes	50 (2.53)	38 (2.32)	9 (4.89)	3 (1.90)	
Corticosteroids, n (%)					0.026
No	1602 (80.99)	1331 (81.36)	155 (84.24)	116 (73.42)	
Yes	376 (19.01)	305 (18.64)	29 (15.76)	42 (26.58)	
Insulin, n (%)					<0.001
No	343 (17.34)	328 (20.05)	7 (3.80)	8 (5.06)	
Yes	1635 (82.66)	1308 (79.95)	177 (96.20)	150 (94.94)	
Dextrose Infusion, n (%)					<0.001
No	279 (14.11)	255 (15.59)	11 (5.98)	13 (8.23)	
Yes	1699 (85.89)	1381 (84.41)	173 (94.02)	145 (91.77)	
**Outcome**					
Hosp Time 28 d	28.00 (25.06, 28.00)	28.00 (28.00,28.00)	28.00 (6.49,28.00)	28.00 (11.10,28.00)	<0.001
Hosp Outcome 28 d, n (%)					
Survival	1472 (74.42)	1262 (77.14)	112 (60.87)	98 (62.03)	
Mortality	506 (25.58)	374 (22.86)	72 (39.13)	60 (37.97)	

SBP, Systolic Blood Pressure; DBP, Diastolic Blood Pressure; MBP, Mean Blood Pressure; Resp Rate, Respiratory Rate; INR, International Normalized Ratio; PT, Prothrombin Time; PTT, Partial Thromboplastin Time; WBC, White Blood Cell; RDW, Red Cell Distribution Width; RBC, Red Blood Cell; MCV, Mean Corpuscular Volume; MCH, Mean Corpuscular Hemoglobin; BUN, Blood Urea Nitrogen; SOFA, Sequential Organ Failure Assessment; SAPSII, Simplified Acute Physiology Score II; GCS, Glasgow Coma Scale; AKI, Acute Kidney Injury; CRRT, Continuous Renal Replacement Therapy.

Univariate Cox regression identified multiple demographic, clinical, and laboratory variables associated with 28-day in-hospital mortality (Table 3). Older age, non-White race, major comorbidities, organ failure, higher illness severity scores, and markers of renal dysfunction, inflammation, and metabolic derangement were associated with increased mortality risk, whereas higher hemoglobin, hematocrit, bicarbonate, and calcium levels were associated with lower risk. Variables with statistical significance and clinical relevance were subsequently considered for multivariable analysis.

**Table 3:** Univariate Cox regression analysis of factors associated with 28-day mortality in ICU patients with intracerebral hemorrhage.

Variables	*P*	HR (95% CI)
Race		
White		1.00 (Reference)
Black	0.886	0.98 (0.72 ~ 1.33)
Others	<0.001	1.54 (1.28 ~ 1.86)
AKI		
No		1.00 (Reference)
Yes	0.033	1.31 (1.02 ~ 1.68)
Intraventricular Hemorrhage		
No		1.00 (Reference)
Yes	<0.001	1.42 (1.15 ~ 1.75)
Renal Disease		
No		1.00 (Reference)
Yes	0.038	1.28 (1.01 ~ 1.61)
Liver Disease		
No		1.00 (Reference)
Yes	<0.001	1.81 (1.36 ~ 2.40)
Sepsis		
No		1.00 (Reference)
Yes	<0.001	1.89 (1.58 ~ 2.26)
Respiratory Failure		
No		1.00 (Reference)
Yes	<0.001	2.65 (2.22 ~ 3.15)
Congestive Heart Failure		
No		1.00 (Reference)
Yes	0.003	1.38 (1.11 ~ 1.72)
Dementia		
No		1.00 (Reference)
Yes	0.002	1.59 (1.18 ~ 2.14)
Ventilator		
No		1.00 (Reference)
Yes	<0.001	1.54 (1.23 ~ 1.93)
CRRT		
No		1.00 (Reference)
Yes	<0.001	2.78 (1.91 ~ 4.04)
Corticosteroids		
No		1.00 (Reference)
Yes	0.001	0.66 (0.52 ~ 0.85)
Dextrose Infusion		
No		1.00 (Reference)
Yes	0.002	1.57 (1.18 ~ 2.11)
Age	<0.001	1.02 (1.01 ~ 1.03)
DBP	0.031	0.99 (0.99 ~ 0.99)
Resp Rate	0.004	1.02 (1.01 ~ 1.04)
SpO_2_	0.027	1.04 (1.01 ~ 1.07)
Potassium	<0.001	1.24 (1.10 ~ 1.39)
Creatinine	<0.001	1.07 (1.04 ~ 1.11)
WBC	0.007	1.01 (1.01 ~ 1.01)
RDW	<0.001	1.12 (1.08 ~ 1.16)
RBC	<0.001	0.73 (0.65 ~ 0.82)
Platelet	<0.001	0.99 (0.99 ~ 0.99)
Hemoglobin	<0.001	0.91 (0.88 ~ 0.95)
Hematocrit	<0.001	0.97 (0.96 ~ 0.98)
MCV	<0.001	1.03 (1.02 ~ 1.04)
INR	<0.001	1.16 (1.07 ~ 1.26)
PT	<0.001	1.01 (1.01 ~ 1.02)
PTT	0.010	1.01 (1.01 ~ 1.01)
Aniongap	<0.001	1.05 (1.03 ~ 1.07)
Bicarbonate	0.025	0.97 (0.95 ~ 0.99)
Calcium	<0.001	0.83 (0.75 ~ 0.91)
BUN	<0.001	1.01 (1.01 ~ 1.02)
SOFA	<0.001	1.20 (1.15 ~ 1.25)
SAPS II	<0.001	1.05 (1.04 ~ 1.05)
Charlson Comorbidity Index	<0.001	1.08 (1.05 ~ 1.11)

HR: Hazard Ratio; CI: Confidence Interval; DBP, Diastolic Blood Pressure; Resp Rate, Respiratory Rate; SpO_2_,Peripheral capillary oxygen saturation; INR, International Normalized Ratio; PT, Prothrombin Time; PTT, Partial Thromboplastin Time; WBC, White Blood Cell; RDW, Red Cell Distribution Width; RBC, Red Blood Cell; MCV, Mean Corpuscular Volume; BUN, Blood Urea Nitrogen; SOFA, Sequential Organ Failure Assessment; SAPSII, Simplified Acute Physiology Score II; AKI, Acute Kidney Injury; CRRT, Continuous Renal Replacement Therapy.

Table 4 shows the association between blood glucose trajectory classes and 28-day mortality in ICU patients with intracerebral hemorrhage under different adjustment models. Using Class 1 as the reference, Class 2 and Class 3 were consistently associated with a significantly higher risk of 28-day mortality across all models. In Model 1 and Model 2, Class 2 showed an approximately twofold increased risk of death, while Class 3 also demonstrated a markedly elevated risk (all *P* < 0.001). After full adjustment in Model 3, the associations remained robust, with Class 2 (HR 1.82, 95% CI 1.39–2.37) and Class 3 (HR 1.70, 95% CI 1.28–2.27) still showing significantly increased mortality risk. These findings indicate that unfavorable blood glucose trajectories are independently associated with higher short-term mortality in patients with intracerebral hemorrhage.

**Table 4:** Hazard ratios for 28-day mortality by blood glucose trajectory classes under different adjustment models in ICU patients with intracerebral hemorrhage.

Class	Model 1		Model 2		Model 3	
	HR (95%CI)	*P*	HR (95%CI)	*P*	HR (95%CI)	*P*
Class 1	Reference		Reference		Reference	
Class 2	2.03 (1.58,2.61)	<0.001	2.06 (1.60,2.66)	<0.001	1.82 (1.39, 2.37)	<0.001
Class 3	1.85 (1.41,2.43)	<0.001	1.85 (1.41,2.43)	<0.001	1.70 (1.28, 2.27)	<0.001

HR, hazard_ratio; CI, confidence_interval.

Model 1: Crude.

Model 2: Adjust:gender, age, race.

Model 3: Adjust:gender, age, race, DBP, resp_rate, SpO_2_, potassium, creatinine, WBC, RDW, RBC, platelet, hemoglobin, hematocrit, mcv, INR, PT, PTT, aniongap, bicarbonate, calcium, BUN, SOFA, SAPSII, charlson_comorbidity_index, AKI, intraventricular_hemorrhage, renal_disease, liver_disease, sepsis, respiratory_failure, congestive_heart_failure, dementia, ventilator, crrt, corticosteroids, dextrose_infusion.

### 3.3. Robustness of the associations across landmark and sensitivity analyses

The associations between early glycemic trajectory classes and 28-day in-hospital mortality were consistent across multiple analytic approaches. In the primary analysis excluding patients with ICU length of stay <36 hours, higher-risk glycemic trajectories were associated with increased mortality. Similar associations were observed in the 36-hour landmark analysis restricted to patients who were alive and still hospitalized at 36 hours after ICU admission (S2 Table). In addition, sensitivity analyses including patients with ICU length of stay <36 hours yielded comparable results, with both Class 2 and Class 3 remaining significantly associated with higher 28-day in-hospital mortality compared with Class 1 (S3 Table). Together, these findings demonstrate that the observed associations were robust and not materially influenced by early deaths, short ICU stays, or analytic assumptions.

### 3.4. Kaplan-Meier analysis of 28-day mortality by glucose trajectory

**Fig 3:**
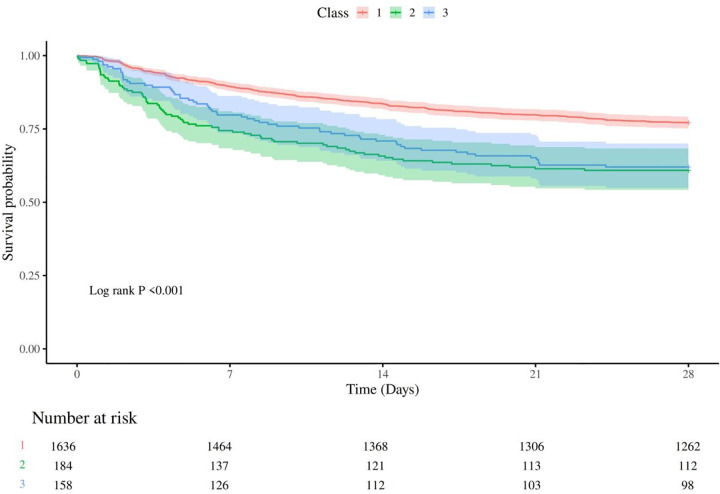
28-day survival curves of ICU patients with intracerebral hemorrhage by blood glucose trajectory classes. The figure shows the survival probabilities over 28 days for three patient subgroups classified by blood glucose trajectories. Patients in Class 1 (red) have the highest survival rate, while those in Class 2 (green) and Class 3 (blue) have lower survival rates. The differences among the three survival curves are statistically significant (Log-rank *P* < 0.001).

Kaplan-Meier survival analysis demonstrated a significant difference in 28-day survival probabilities among the blood glucose trajectory classes (Log-rank test, *P* < 0.001). Patients in Class 1 exhibited the highest survival rate, with a survival curve consistently above those of Classes 2 and 3. Classes 2 and 3 showed lower and closely overlapping survival probabilities, indicating poorer prognosis in these groups. The number of patients at risk decreased over the follow-up period in all classes, with Class 1 maintaining higher numbers throughout. These findings further support the prognostic value of blood glucose trajectories in patients with intracerebral hemorrhage (Fig 3).

### 3.5. Subgroup analysis of blood glucose trajectories and mortality in intracerebral hemorrhage

**Fig 4:**
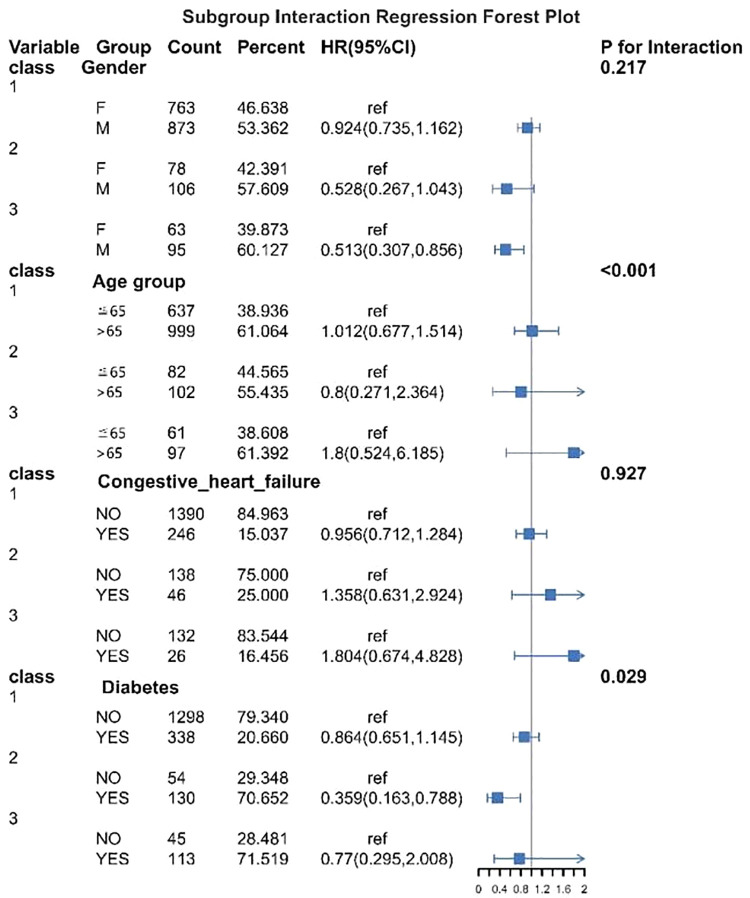
Forest plot of subgroup interactions between clinical variables and blood glucose trajectory classes on 28-day mortality in ICU patients with intracerebral hemorrhage. This forest plot illustrates hazard ratios (HR) and 95% confidence intervals (CI) for subgroup variables including gender, age group, congestive heart failure, and diabetes across different blood glucose trajectory classes (class 1–3).

Fig 4 shows the subgroup interaction analyses of the association between glucose trajectory classes and 28-day in-hospital mortality. No significant interactions were observed for sex (P for interaction = 0.217) or congestive heart failure (*P* = 0.927). Significant interactions were detected for age group (*P* < 0.001) and diabetes status (*P* = 0.029), indicating that the association between glucose trajectories and mortality varied across these subgroups.

## 4. Discussion

In this ICU cohort of patients with intracerebral hemorrhage, we identified three distinct early blood glucose trajectory patterns within the first 36 hours after ICU admission. Compared with patients with stable glucose levels, those with unfavorable trajectories exhibited worse clinical characteristics and significantly higher 28-day in-hospital mortality. These associations remained consistent across multivariable models, a 36-hour landmark analysis, and sensitivity analyses including patients with short ICU stays. Kaplan–Meier and subgroup analyses further supported the prognostic value of early glucose trajectories, with effect modification observed by age and diabetes status.

Blood glucose trajectory, as a dynamic biomarker, provides a more comprehensive reflection of metabolic changes throughout the disease course in patients with severe intracerebral hemorrhage compared to single-point glucose measurements. A one-time glucose reading may be influenced by various factors such as stress responses and therapeutic interventions, making it difficult to accurately assess the patient’s true metabolic state.In contrast, trajectory can reveal trends and patterns of glucose variation, offering insights into the body’s capacity for metabolic regulation [14,15]. Studies have shown that sustained hyperglycemia or highly fluctuating trajectory are closely associated with enhanced inflammatory responses, blood-brain barrier disruption, and poor neurological recovery [16–18]. Analysis of blood trajectory can facilitate more effective risk stratification and personalized management, providing dynamic and precise decision support for clinical practice.

In this study, clustering analysis based on blood glucose trajectory identified three representative dynamic patterns. Class 1 exhibited overall stable glucose levels maintained within the normal range, indicating good metabolic control and stress response capacity, which may be associated with more favorable clinical outcomes. Class 2 showed a typical U-shaped trajectory—initial hyperglycemia, followed by a decline, and then a subsequent rise—reflecting significant glucose fluctuations. This pattern may indicate a sustained stress state or metabolic imbalance, potentially exerting negative effects on neurological recovery. Class 3 presented an inverted U-shaped trajectory, characterized by elevated glucose levels in the early phase followed by a decline in the later phase. This may represent a subgroup with initially high metabolic burden that was partially controlled over time, suggesting an intermediate-risk population. These distinct trajectory patterns reflect, to some extent, individual differences in metabolic responses under critical illness, further supporting the clinical value of blood glucose trajectory as dynamic prognostic biomarkers.

The underlying mechanisms of blood glucose variability involve a range of complex physiological and pathological processes, primarily including oxidative stress, inflammatory responses, endothelial dysfunction, and autonomic nervous system dysregulation. First, numerous studies have shown that rapid fluctuations in glucose levels are more likely to trigger oxidative stress than sustained hyperglycemia. Sudden increases or decreases in glucose can activate NADPH oxidase, leading to elevated production of reactive oxygen species (ROS), which in turn damages cellular structures and functions [18,19]. Second, blood glucose variability can induce an inflammatory cascade by activating the NF-κB signaling pathway, which promotes the release of pro-inflammatory cytokines such as IL-6 and TNF-α. This process leads to endothelial damage in both microvascular and macrovascular systems and accelerates the development of atherosclerosis [20]. Additionally, frequent alternation between hyperglycemia and hypoglycemia can impair endothelial nitric oxide (NO) production, weaken vasodilation function, exacerbate vascular tone abnormalities, and increase the risk of cardiovascular events [19]. In critically ill conditions, especially in patients with acute brain injuries such as intracerebral hemorrhage, blood glucose variability may also reflect dysregulated central nervous system responses to stress.

Activation of the hypothalamic-pituitary-adrenal (HPA) axis and sympathetic nervous system stimulation can both affect glucose metabolism, resulting in highly unstable patterns of blood glucose fluctuations [21]. In summary, blood glucose variability is not only a marker of metabolic dysregulation but may also serve as a driving factor in various pathological processes. Its underlying mechanisms involve oxidative stress, inflammatory injury, endothelial dysfunction, and neuroendocrine dysregulation, providing a biological basis for its detrimental role in disease progression and prognosis.Consistent with findings from studies on other critical illnesses, blood glucose trajectory have been confirmed as important prognostic indicators in patients with severe conditions such as brain injury [22]. Studies have shown that dynamic patterns of blood glucose change better reflect an individual’s pathological state and metabolic response than static glucose values, aiding in the early identification of high-risk patients. This study offers a novel exploration of blood glucose trajectory in patients with intracerebral hemorrhage, contributing to the growing body of research in this area.

In the subgroup analysis of this study, both age and diabetic status showed significant interactions with blood glucose trajectory (P for interaction < 0.001 and 0.029, respectively), suggesting that the impact of blood trajectory on prognosis may vary across different populations. Specifically, older patients may have reduced tolerance to glucose fluctuations due to diminished metabolic reserves, impaired autonomic function, and increased insulin resistance, making them more susceptible to the effects of unfavorable blood glucose trajectory. In addition, elderly individuals often present with multiple chronic conditions and a pro-inflammatory state, which may synergize with abnormal glucose variability to exacerbate brain tissue damage and functional deterioration [23–25]. Diabetic status is also a key interacting factor. Diabetic patients inherently exhibit impaired β-cell function, insulin resistance, and a chronic inflammatory background, making their glucose regulation mechanisms more fragile and their adaptability to glucose fluctuations weaker. Abnormal glucose trajectory in this population may trigger more intense oxidative stress, endothelial dysfunction, and microcirculatory disturbances, thereby exerting a greater negative impact on neurological recovery [26,27]. In contrast, non-diabetic patients may have a greater compensatory capacity to tolerate blood glucose fluctuations within a certain range.

In observational ICU studies, excluding patients with short ICU stays may introduce survivor bias and immortal time bias. To mitigate these biases, we applied a 36-hour landmark analysis, restricting the study population to patients who were alive and still hospitalized at 36 hours after ICU admission. Glucose trajectories were defined exclusively based on glucose measurements obtained within the first 36 hours after ICU admission, ensuring that exposure classification preceded the start of follow-up and thereby methodologically reducing the risk of immortal time bias.In addition, a sensitivity analysis was conducted by including patients with an ICU length of stay shorter than 36 hours. The consistency in the direction and magnitude of effect estimates across the primary analysis, landmark analysis, and sensitivity analysis indicates that the association between early glucose trajectories and 28-day in-hospital mortality is robust and unlikely to be driven by early deaths or short ICU stays.

This study has several strengths. First, it included a relatively large sample size, which enhanced the statistical power and representativeness of the findings. Second, a wide range of potential confounders was adjusted for using multivariable models, improving the robustness of the conclusions. In addition, the use of blood glucose trajectories as dynamic indicators provided a more comprehensive reflection of patients’ metabolic status and temporal trends compared with traditional single time-point glucose measurements.However, several limitations should be acknowledged. Due to the retrospective observational design, selection bias and information bias cannot be completely excluded. The data were derived from a single center or a specific database, which may limit the generalizability of the results. Although intraventricular hemorrhage (IVH) status was included as a covariate in the analysis, other important ICH-specific severity indicators—such as hematoma volume, hemorrhage location, and detailed neurosurgical interventions—were not comprehensively available in the database. While global severity scores (SOFA and SAPS II) were adjusted for, residual confounding related to ICH severity may still exist. In addition, the frequency of blood glucose monitoring in routine clinical practice was limited, which may have affected the precision of glucose trajectory modeling.

## 5. Conclusion

Our study demonstrates that blood glucose trajectory is a significant prognostic marker in patients with intracerebral hemorrhage. Distinct glycemic patterns identified within the early ICU period are strongly associated with 28-day mortality, independent of other clinical factors. These findings highlight the importance of continuous glucose monitoring and suggest that incorporating glucose trajectory analysis into clinical practice could improve outcome prediction and tailor therapeutic interventions. Future prospective studies are warranted to validate these results and explore optimal glucose control strategies in this high-risk population.

## Supporting information

S1 Table Baseline characteristics of ICU patients with intracerebral hemorrhage stratified by 28-day survival status. This table provides a detailed comparison of demographic and clinical characteristics between survivors and non-survivors at 28 days.
(DOCX)

S2 Table Associations between glycemic trajectory classes and 28-day in-hospital mortality in the 36-hour landmark cohort. This table presents the results of the primary analysis using the landmark approach to evaluate mortality risk across different glycemic patterns.
(DOCX)

S3 Table Sensitivity analysis of glycemic trajectory classes and 28-day in-hospital mortality. This table includes various sensitivity analyses to confirm the robustness of the primary findings.
(DOCX)
