# Supplementary material for: Prognostic value of blood glucose trajectories in critically ill patients with intracerebral hemorrhage: A retrospective cohort study
Source: PLoS One. 2026 Feb 24;21(2):e0342745. doi: 10.1371/journal.pone.0342745 (PMC12931793; doi:10.1371/journal.pone.0342745)
Supplement: S1 Table — This table provides a detailed comparison of demographic and clinical characteristics between survivors and non-survivors at 28 days. (DOCX) [file pone.0342745.s001.docx]

Supplementary Table 1. Baseline Characteristics of ICU Patients With Intracerebral Hemorrhage Stratified by 28-Day Survival Status

| Variables | Total (n = 1978) | Survival (n = 1472) | Mortality (n = 506) | *P* |
| --- | --- | --- | --- | --- |
|  |  |  |  |  |
| Age | 69.45 (57.95, 79.95) | 67.88 (56.95, 78.46) | 73.72 (62.24, 82.88) | <0.001 |
| Gender, n(%) |  |  |  | 0.266 |
| Female | 904 (45.70) | 662 (44.97) | 242 (47.83) |  |
| Man | 1074 (54.30) | 810 (55.03) | 264 (52.17) |  |
| Race, n(%) |  |  |  | <0.001 |
| White | 1146 (57.94) | 886 (60.19) | 260 (51.38) |  |
| Black | 214 (10.82) | 166 (11.28) | 48 (9.49) |  |
| Others | 618 (31.24) | 420 (28.53) | 198 (39.13) |  |
| Class, n(%) |  |  |  | <0.001 |
| 1 | 1636 (82.71) | 1262 (85.73) | 374 (73.91) |  |
| 2 | 184 (9.30) | 112 (7.61) | 72 (14.23) |  |
| 3 | 158 (7.99) | 98 (6.66) | 60 (11.86) |  |
| Vital signs |  |  |  |  |
| Heart Rate,bpm | 81.50 (71.00, 94.00) | 81.00 (71.00, 93.00) | 82.50 (70.25, 95.75) | 0.300 |
| SBP,mmHg | 137.00 (122.00, 151.00) | 137.00 (123.00, 150.00) | 137.00 (120.00, 152.75) | 0.794 |
| DBP,mmHg | 74.00 (64.00, 86.00) | 74.00 (64.00, 86.00) | 72.00 (61.00, 85.00) | 0.021 |
| MBP,mmHg | 91.00 (81.00, 102.75) | 91.00 (82.00, 103.00) | 91.00 (78.00, 102.00) | 0.065 |
| Resp Rate,bpm | 18.00 (15.50, 22.00) | 18.00 (15.00, 21.00) | 19.00 (16.00, 22.00) | 0.002 |
| Temperature,℃ | 36.83 (36.52, 37.11) | 36.83 (36.56, 37.11) | 36.75 (36.44, 37.17) | 0.054 |
| SpO_2_,% | 98.00 (96.00, 100.00) | 98.00 (96.00, 100.00) | 99.00 (96.00, 100.00) | <0.001 |
| Laboratory index |  |  |  |  |
| Sodium,mEq/L | 139.00 (137.00, 142.00) | 139.00 (137.00, 142.00) | 139.00 (136.25, 142.00) | 0.582 |
| Potassium,mEq/L | 3.90 (3.60, 4.30) | 3.90 (3.60, 4.30) | 4.00 (3.60, 4.40) | 0.001 |
| Creatinine,mEq/L | 0.90 (0.70, 1.10) | 0.90 (0.70, 1.10) | 1.00 (0.70, 1.30) | <0.001 |
| WBC,K/uL | 10.50 (8.10, 13.50) | 10.20 (7.90, 13.10) | 11.20 (9.00, 14.97) | <0.001 |
| RDW,% | 13.90 (13.10, 14.90) | 13.70 (13.00, 14.70) | 14.20 (13.40, 15.40) | <0.001 |
| RBC,M/uL | 4.07 (3.60, 4.52) | 4.12 (3.65, 4.56) | 3.91 (3.41, 4.38) | <0.001 |
| Platelet,K/uL | 208.00 (160.00, 259.00) | 211.00 (165.00, 261.00) | 192.50 (141.00, 250.00) | <0.001 |
| Hemoglobin,g/dL | 12.20 (10.80, 13.50) | 12.30 (10.90, 13.62) | 11.70 (10.22, 13.20) | <0.001 |
| Hematocrit,% | 36.75 (32.82, 40.40) | 37.10 (33.27, 40.70) | 35.60 (31.70, 39.58) | <0.001 |
| MCV,fL | 30.20 (28.70, 31.60) | 30.10 (28.60, 31.50) | 30.30 (28.90, 31.80) | 0.101 |
| MCH,pg | 91.00 (87.00, 95.00) | 91.00 (87.00, 94.00) | 92.00 (88.00, 96.00) | <0.001 |
| INR | 1.10 (1.10, 1.30) | 1.10 (1.10, 1.20) | 1.20 (1.10, 1.40) | <0.001 |
| PT | 12.60 (11.70, 14.10) | 12.50 (11.60, 13.70) | 13.10 (12.00, 15.10) | <0.001 |
| PTT | 28.30 (25.80, 31.40) | 28.30 (25.80, 31.13) | 28.80 (25.70, 33.00) | 0.030 |
| Chloride,mEq/L | 104.00 (101.00, 107.00) | 104.00 (101.00, 106.00) | 103.00 (100.00, 107.00) | 0.201 |
| Aniongap,mEq/L | 14.00 (12.00, 16.00) | 14.00 (12.00, 16.00) | 15.00 (13.00, 17.00) | <0.001 |
| Bicarbonate,mEq/L | 23.00 (21.00, 25.00) | 23.00 (21.00, 25.00) | 23.00 (20.00, 25.00) | 0.012 |
| Calcium,mEq/L | 8.80 (8.30, 9.20) | 8.80 (8.40, 9.20) | 8.70 (8.20, 9.10) | <0.001 |
| BUN,mg/dL | 16.00 (12.00, 23.00) | 16.00 (12.00, 21.00) | 19.00 (13.00, 27.00) | <0.001 |
| Score |  |  |  |  |
| SOFA | 1.00 (0.00, 2.00) | 0.00 (0.00, 1.00) | 1.00 (0.00, 2.00) | <0.001 |
| SAPSII | 33.00 (26.00, 41.00) | 31.00 (25.00, 38.00) | 40.00 (33.00, 48.00) | <0.001 |
| GCS | 14.00 (11.00, 15.00) | 14.00 (12.00, 15.00) | 14.00 (9.00, 15.00) | 0.700 |
| Charlson Comorbidity Index | 6.00 (4.00, 8.00) | 6.00 (4.00, 8.00) | 7.00 (5.00, 8.00) | <0.001 |
| Complication |  |  |  |  |
| AKI, n(%) |  |  |  | 0.021 |
| No | 348 (17.59) | 276 (18.75) | 72 (14.23) |  |
| Yes | 1630 (82.41) | 1196 (81.25) | 434 (85.77) |  |
| Intraventricular Hemorrhage, n(%) |  |  |  | 0.002 |
| No | 1620 (81.90) | 1229 (83.49) | 391 (77.27) |  |
| Yes | 358 (18.10) | 243 (16.51) | 115 (22.73) |  |
| Myocardial Infarct, n(%) |  |  |  | 0.055 |
| No | 1790 (90.50) | 1343 (91.24) | 447 (88.34) |  |
| Yes | 188 (9.50) | 129 (8.76) | 59 (11.66) |  |
| Renal Disease, n(%) |  |  |  | 0.026 |
| No | 1697 (85.79) | 1278 (86.82) | 419 (82.81) |  |
| Yes | 281 (14.21) | 194 (13.18) | 87 (17.19) |  |
| Liver Disease, n(%) |  |  |  | <0.001 |
| No | 1845 (93.28) | 1393 (94.63) | 452 (89.33) |  |
| Yes | 133 (6.72) | 79 (5.37) | 54 (10.67) |  |
| Sepsis, n(%) |  |  |  | <0.001 |
| No | 1020 (51.57) | 831 (56.45) | 189 (37.35) |  |
| Yes | 958 (48.43) | 641 (43.55) | 317 (62.65) |  |
| Respiratory Failure, n(%) |  |  |  | <0.001 |
| No | 1306 (66.03) | 1075 (73.03) | 231 (45.65) |  |
| Yes | 672 (33.97) | 397 (26.97) | 275 (54.35) |  |
| Congestive Heart Failure, n(%) |  |  |  | 0.002 |
| No | 1660 (83.92) | 1257 (85.39) | 403 (79.64) |  |
| Yes | 318 (16.08) | 215 (14.61) | 103 (20.36) |  |
| Peripheral Vascular Disease, n(%) |  |  |  | 0.858 |
| No | 1833 (92.67) | 1365 (92.73) | 468 (92.49) |  |
| Yes | 145 (7.33) | 107 (7.27) | 38 (7.51) |  |
| Dementia, n(%) |  |  |  | 0.001 |
| No | 1850 (93.53) | 1392 (94.57) | 458 (90.51) |  |
| Yes | 128 (6.47) | 80 (5.43) | 48 (9.49) |  |
| Chronic Pulmonary Disease, n(%) |  |  |  | 0.440 |
| No | 1705 (86.20) | 1274 (86.55) | 431 (85.18) |  |
| Yes | 273 (13.80) | 198 (13.45) | 75 (14.82) |  |
| Rheumatic Disease, n(%) |  |  |  | 0.988 |
| No | 1927 (97.42) | 1434 (97.42) | 493 (97.43) |  |
| Yes | 51 (2.58) | 38 (2.58) | 13 (2.57) |  |
| Peptic Ulcer Disease, n(%) |  |  |  | 0.359 |
| No | 1962 (99.19) | 1458 (99.05) | 504 (99.60) |  |
| Yes | 16 (0.81) | 14 (0.95) | 2 (0.40) |  |
| Diabetes, n(%) |  |  |  | 0.198 |
| No | 1397 (70.63) | 1051 (71.40) | 346 (68.38) |  |
| Yes | 581 (29.37) | 421 (28.60) | 160 (31.62) |  |
| Hypertension, n(%) |  |  |  | 0.436 |
| No | 726 (36.70) | 533 (36.21) | 193 (38.14) |  |
| Yes | 1252 (63.30) | 939 (63.79) | 313 (61.86) |  |
| Treatment |  |  |  |  |
| Ventilator, n(%) |  |  |  | <0.001 |
| No | 508 (25.68) | 413 (28.06) | 95 (18.77) |  |
| Yes | 1470 (74.32) | 1059 (71.94) | 411 (81.23) |  |
| Crrt, n(%) |  |  |  | <0.001 |
| No | 1928 (97.47) | 1451 (98.57) | 477 (94.27) |  |
| Yes | 50 (2.53) | 21 (1.43) | 29 (5.73) |  |
| Corticosteroids, n(%) |  |  |  | 0.002 |
| No | 1602 (80.99) | 1169 (79.42) | 433 (85.57) |  |
| Yes | 376 (19.01) | 303 (20.58) | 73 (14.43) |  |
| Insulin, n(%) |  |  |  | 0.185 |
| No | 343 (17.34) | 265 (18.00) | 78 (15.42) |  |
| Yes | 1635 (82.66) | 1207 (82.00) | 428 (84.58) |  |
| Dextrose Infusion, n(%) |  |  |  | 0.002 |
| No | 279 (14.11) | 229 (15.56) | 50 (9.88) |  |
| Yes | 1699 (85.89) | 1243 (84.44) | 456 (90.12) |  |
