# Supplementary material for: Prognostic value of blood glucose trajectories in critically ill patients with intracerebral hemorrhage: A retrospective cohort study
Source: PLoS One. 2026 Feb 24;21(2):e0342745. doi: 10.1371/journal.pone.0342745 (PMC12931793; doi:10.1371/journal.pone.0342745)
Supplement: S2 Table — This table presents the results of the primary analysis using the landmark approach to evaluate mortality risk across different glycemic patterns. (DOCX) [file pone.0342745.s002.docx]

Supplementary Table 2. Associations between glycemic trajectory classes and 28-day in-hospital mortality in the 36-hour landmark cohort

| Class | Model1 | | Model2 | | Model3 | |
| --- | --- | --- | --- | --- | --- | --- |
|  | HR(95%CI) | *P* | HR(95%CI) | *P* | HR(95%CI) | *P* |
| Class1 | Reference |  | Reference |  | Reference |  |
| Class2 | 1.76 (1.34, 2.32) | <0.001 | 1.77 (1.35, 2.33) | <0.001 | 1.70 (1.28, 2.25) | <0.001 |
| Class3 | 1.79 (1.34, 2.38) | <0.001 | 1.79 (1.35, 2.38) | <0.001 | 1.51 (1.12, 2.05) | 0.007 |

HR,hazard_ratio;CI,confidence_interval.

Model1:Crude

Model2:Adjust:gender,age,race

Model3:Adjust:gender,age,race,DBP,resp_rate,SpO_2_,potassium,creatinine,WBC,RDW,RBC,platelet,hemoglobin,hematocrit,MCV,INR,PT,PTT,aniongap,bicarbonate,calcium,BUN,SOFA,SAPSII,charlson_comorbidity_index,AKI,intraventricular_hemorrhage,renal_disease,liver_disease,sepsis,respiratory_failure,congestive_heart_failure,dementia,ventilator,crrt,corticosteroids,dextrose_infusion
