# Supplementary material for: Prognostic value of blood glucose trajectories in critically ill patients with intracerebral hemorrhage: A retrospective cohort study
Source: PLoS One. 2026 Feb 24;21(2):e0342745. doi: 10.1371/journal.pone.0342745 (PMC12931793; doi:10.1371/journal.pone.0342745)
Supplement: S3 Table — This table includes various sensitivity analyses to confirm the robustness of the primary findings. (DOCX) [file pone.0342745.s003.docx]

Supplementary Table 3. Sensitivity analysis of glycemic trajectory classes and 28-day in-hospital mortality

| Class | Model1 | | Model2 | | Model3 | |
| --- | --- | --- | --- | --- | --- | --- |
|  | HR(95%CI) | *P* | HR(95%CI) | *P* | HR(95%CI) | *P* |
| Class1 | Reference |  | Reference |  | Reference |  |
| Class2 | 2.01 (1.57, 2.58) | <0.001 | 2.04 (1.59, 2.61) | <0.001 | 1.92 (1.49, 2.49) | <0.001 |
| Class3 | 1.87 (1.43, 2.44) | <0.001 | 1.87 (1.43, 2.44) | <0.001 | 1.69 (1.27, 2.25) | <0.001 |

HR,hazard_ratio;CI,confidence_interval.

Model1:Crude

Model2:Adjust:gender,age,race

Model3:Adjust:gender,age,race,DBP,resp_rate,SpO_2_,potassium,creatinine,WBC,RDW,RBC,platelet,hemoglobin,hematocrit,MCV,INR,PT,PTT,aniongap,bicarbonate,calcium,BUN,SOFA,SAPSII,charlson_comorbidity_index,AKI,intraventricular_hemorrhage,renal_disease,liver_disease,sepsis,respiratory_failure,congestive_heart_failure,dementia,ventilator,crrt,corticosteroids,dextrose_infusion
